# Supplementary material for: Complex hazard cascade culminating in the Anak Krakatau sector collapse
Source: Nat Commun. 2019 Oct 1;10:4339. doi: 10.1038/s41467-019-12284-5 (PMC6773710; doi:10.1038/s41467-019-12284-5)
Supplement: Supplementary file 1 — Supplementary Information [file 41467_2019_12284_MOESM1_ESM.pdf]

Supplementary Information

Complex hazard cascade culminating in the Anak Krakatau sector collapse

Thomas R. Walter, Mahmud Haghshenas Haghighi, Felix M. Schneider, Diego Coppola, Mahdi Motagh, Joachim Saul, Andrey Babeyko, Torsten Dahm, Valentin R. Troll, Frederik Tilmann, Sebastian Heimann, Sébastien Valade, Rahmat Triyono, Rokhis Khomarudin, Nugraha Kartadinata, Marco Laiolo, Francesco Massimetti, Peter Gaebler

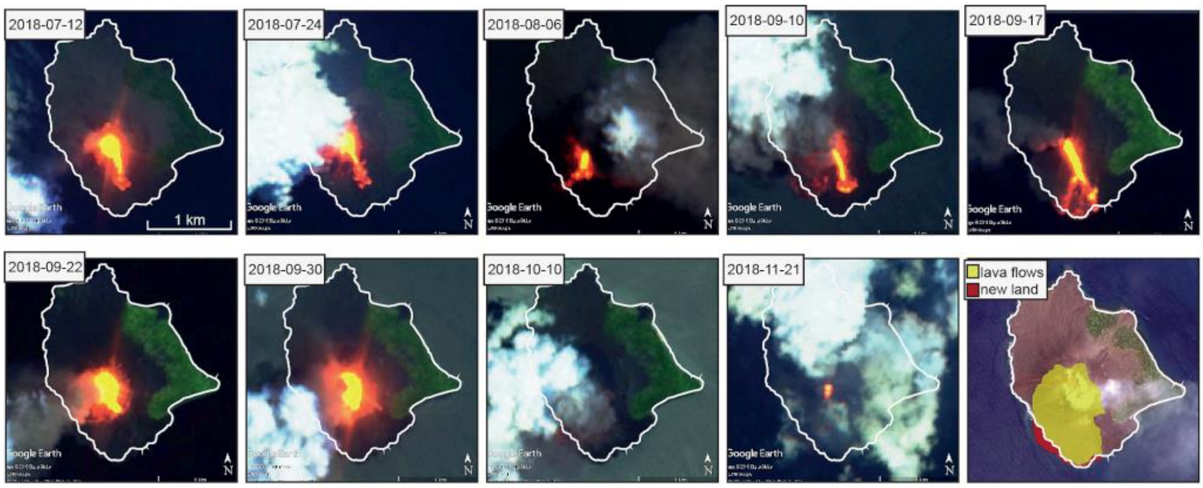

Supplementary Figure 1. Thermal emissions recorded by satellite data. Visual summary of the total extent of thermal anomalies, as measured from the Sentinel 2 images (band combination 12,11,4) showing the emplacement of several hot and new material depositions on the southwestern flank of Anak Krakatau during July-December 2018 (Sentinel 2 images freely available from Copernicus Open Access Hub and retrieved by EO browser Sentinel Hub portal). Whether these hot materials are due to lava flows or due to clastic deposition cannot deduced from this data. The approximate extension of land inundated by new lava flows and/or covered by hot ejecta (~0.85 km^2), is shown on the lower right panel.

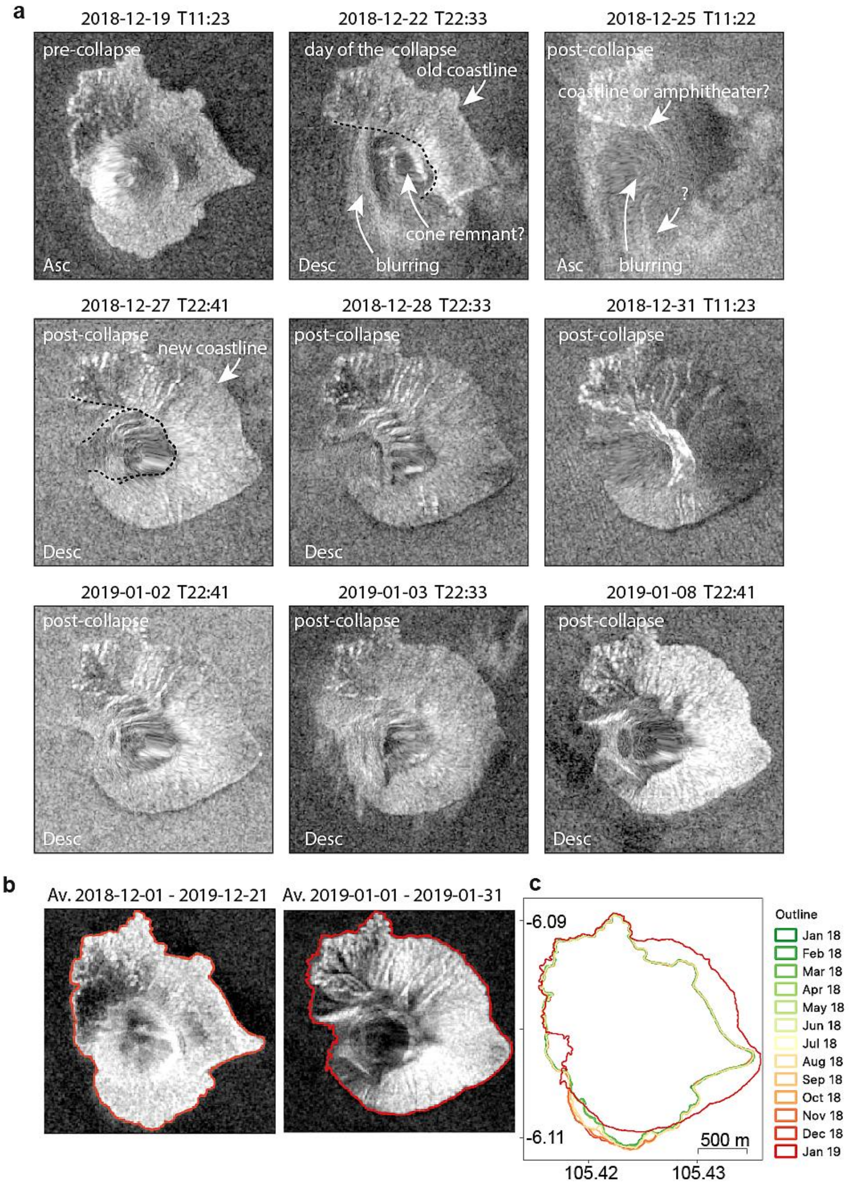

Supplementary Figure 2. Island perimeter monitoring. a Selected Sentinel 1 GRD images before the collapse, on the day of the collapse (the collapse was at 13:55 UTC, the GRD image at 22:33 UTC) and afterwards. Arising from higher radar reflectivity, steep cliffs of landslide amphitheater can be partly identified in the 22 Dec 2018 image (dashed black line). On 25 Dec 2018, the southwest sector blurs due to eruption plume affecting the radar path[1,2]. The image on 27 Dec 2018 reveals the complete amphitheater geometry (partially infilled in the northwest) and the new coastline (shifted by up to 260 m) in the northeast. Dashed black lines in second and fourth image represent approximate outlines of satellite headwalls, which might indicate two outlines, enclosing approximately 0.63km² and 0.84km², which is 44% and 58% of the deformation area identified in InSAR time series, respectively. **b** An island perimeter detection algorithm was used to determine the growth of Anak Krakatau from 1 Jan 2018 to 31 Jan 2019. We utilized the Google Earth Engine cloud computing environment[3] by first computing monthly stacks from the Sentinel 1 ground range detected (GRD) scenes to reduce the speckle and then segmenting the stacked images using an adaptive threshold to separate land from water bodies. The figure shows the examples of Dec 2018 and Jan 2019, for which the stacking has been done to overcome the speckle effect at the cost of averaging short-term variations in the coastline. Each stack considers all Sentinel-1A/B GRD images, ascending and descending, acquired from the first to the last days of a month, except for December 2018, for which GRD images only until December 21 (before the sector collapse) were considered. The enlarged views of the backscatter channel of the GRD images in December 2018 and January 2019 and the average images used to delineate the outlines indicated by red polygons. **c** Monthly evolution of the coastline of Anak Krakatau. Note that largest changes on the southern flank occurred during June-August 2018.

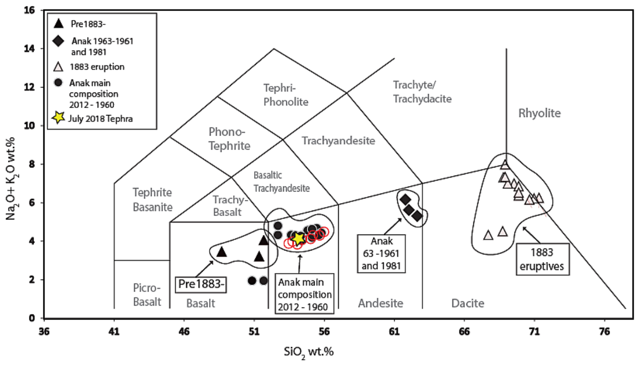

Supplementary Figure 3. Rock analysis. Representative samples of precollapse eruption products were taken from the active flanks (southwestern to northern sectors) of the Anak Krakatau edifice by observatory personnel of the Geological Agency of Indonesia between 2010 and 2018 (red circles), i.e., before the 22 December 2018 flank collapse. This figure shows the total alkali versus silica (TAS) diagram, indicative for the SiO_2 vs Na_2O+$K_2$0 relation for ash and rock samples collected from the stratigraphy corresponding to deposition in 1883, 1960-2012 and 2018 (the red circles and the yellow star represent data from this study). The yellow star sample was from the July 2018 eruption; other data were obtained from previous studies[4,5] and references therein. The reveals that the July 2018 composition was not significantly different than the compositions associated with recent eruptions of Anak Krakatau (e.g., from 1982 to 2012), which are dominantly characterized by basaltic-andesitic bulk compositions. On the other hand, the eruption from 1883 produced dacitic to rhyolitic rocks. Therefore, no indication exists for a deep magmatic change prior to the 2018 activities and the tsunami disaster.

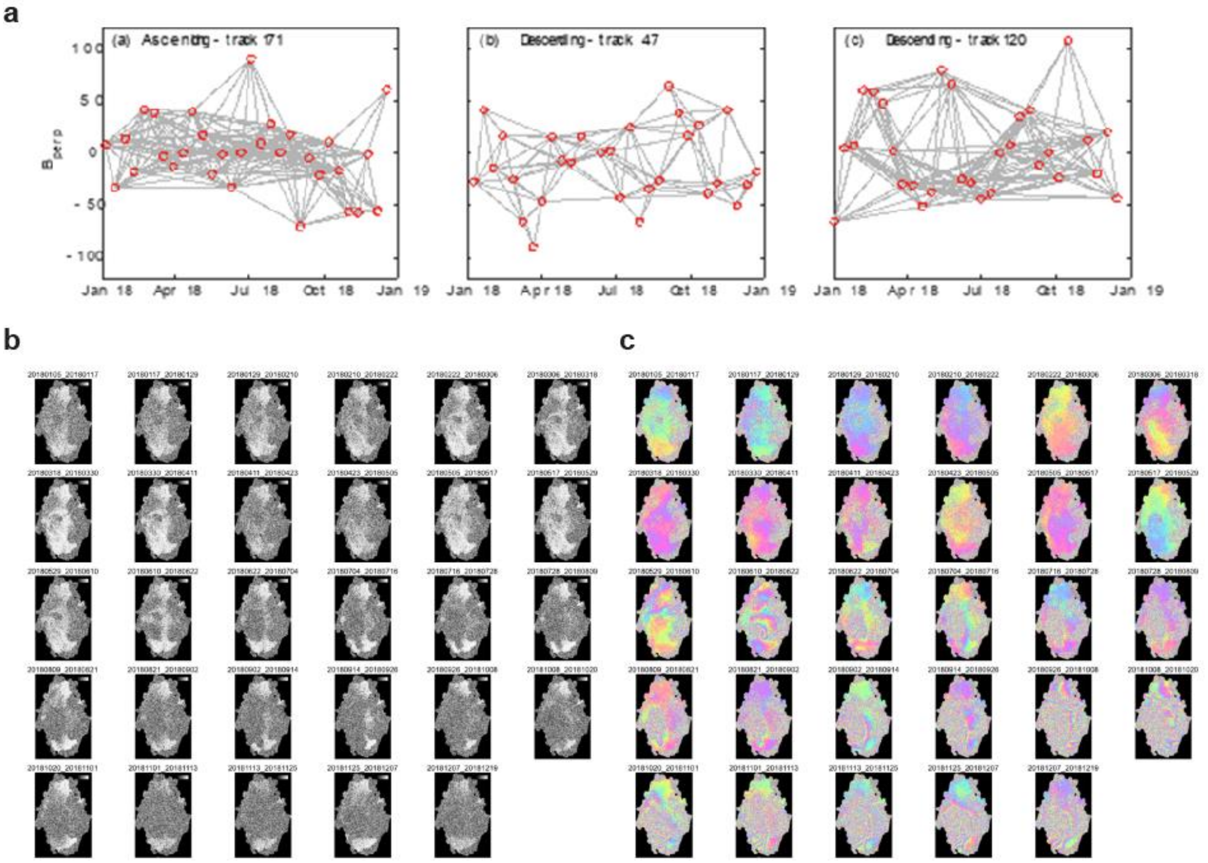

Supplementary Figure 4. Data used in InSAR time series analysis. a Time (month and year) versus the perpendicular baseline (B_perp) of the relevant acquisitions. All data from the Sentinel 1 A/B satellites acquired during 2018 along three different tracks were downloaded and interferometrically processed using StaMPS/MTI, which combines the persistent scatterer and small baseline subset approaches[6]. The topographic component of the interferograms was removed using the 12-meter TanDEM-X DEM. **b** Coherence maps of consecutive SAR images provide an assessment of the location of new volcanic deposits. Areas with dark pixels show low coherence, and areas with bright pixels show high coherence. Note the development of high coherence in the southern sector of the island in the second half of 2018, corresponding to bare rock surfaces of newly emplaced material. These new materials first cause a loss of coherence in the InSAR data after June but then become highly coherent after October. **C** Example ascending-track interferograms used for the generation of an InSAR time series. Only interferograms between consecutive dates are shown in this figure (similar in b). Each color cycle corresponds to 2.8 cm of displacement in the satellite line-of-sight direction. The interferograms before May 2018 only show minor displacement. A clear pattern of displacement is observed after June 2018. Those interferograms spanning a two-pass in the second half of the year 2018 show more clear fringe signals, implying that the deformation trend may have increased. We note that part of the fringe signal observed in the south of the island may be due to loading, compaction and cooling of fresh erupted deposits, thus overprinting the long-term signal, especially in the interferograms that are generated for the period of high effusive activity (after July 2018). As these areas keep coherent in the time series, we speculate that the deposition area still hosts many points that do not change their electromagnetic reflectivity property, meaning that they are not covered.

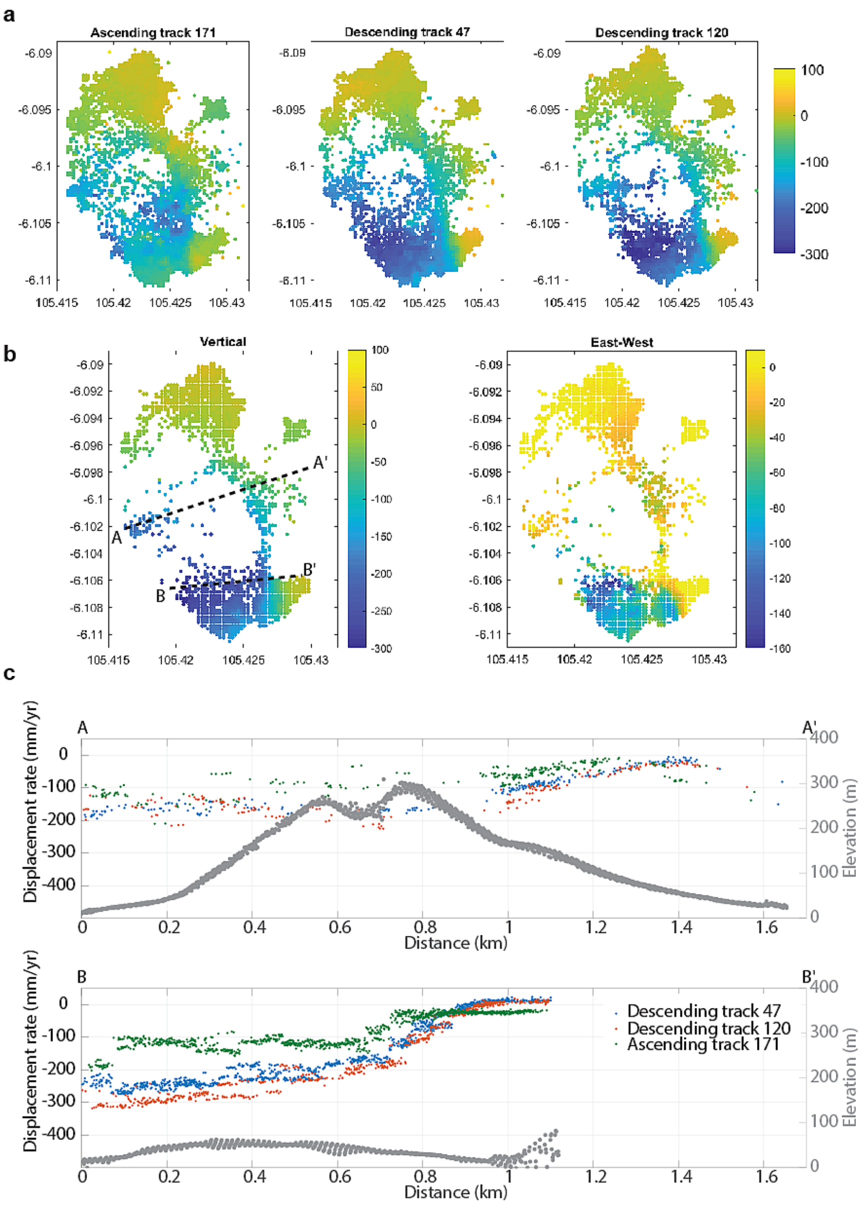

Supplementary Figure 5. InSAR time series and signal decomposition. a The LOS ground motion was estimated, and maps were produced for the period between 1 January and 22 December 2018 by comparing and combining different viewing geometries of one ascending and two descending tracks. **b** The different viewing geometries were combined to calculate the vertical and horizontal motion (in the E-W direction) before the eruption [7]. The chosen reference point is located on the stable northern flank of the island. Color scale in mm/yr. Based on the viewing angles, it was not possible to reconstruct deformation in the N-S direction with acceptable precision. Note location of profiles A-A' and B-B' (black dashed lines). **c** Profiles show comparison of (precollapse) topography versus deformation trend. This figure reveals that the actual deformation is not related to topographic artifacts. Jumps in the profiles close to the coast should not be over interpreted and may result from errors during processing, in the coastline morphology or others. Jumps in the profile B-B' may arise from localized and small scale faulting or from new deposited eruption material.

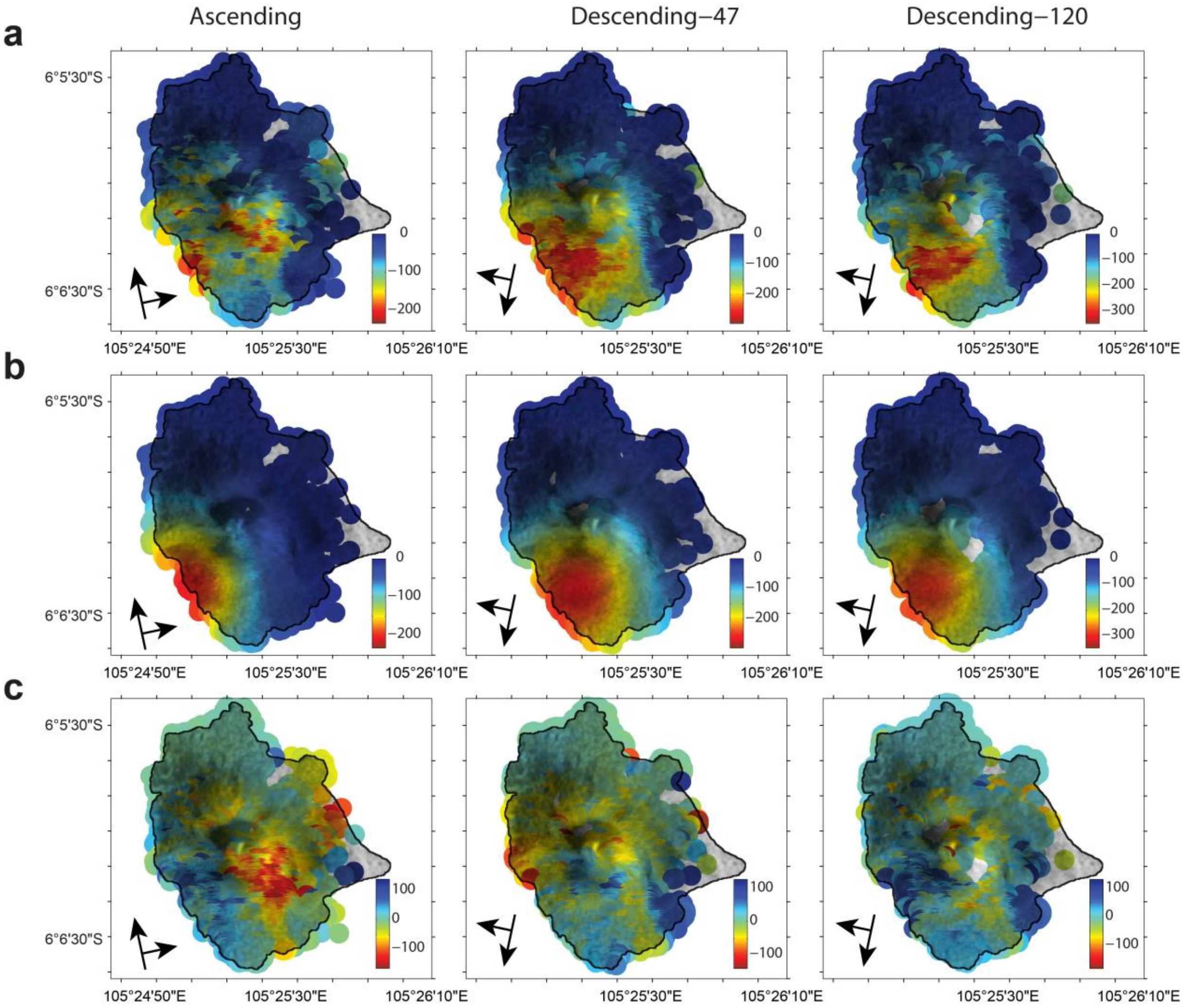

Supplementary Figure 6. InSAR deformation modelling. a. Unwrapped mean line of sight (LOS) velocity data for the investigated tracks and viewing geometries, used for modelling[8]. Landslides may include a plastic flow rheology[9], but may be modelled using elastic dislocations especially for slow-moving landslides[10], in order to assess the geometric and dynamic parameters of the landslide detachment plane[8,11,12]. **b** We use the rectangular dislocation (RD[13]) to search for the source of deformation. The RD is based on the angular dislocation in a half-space[14] and provides geometrical parameters allowing to assess shear and tensile faults in an elastic medium[15,16]. Parameters that determine the orientation of the RD plane are described by the strike angle (α) and the dip angle (δ), while the 'plunge angle' (θ) is the angle between the upper edge of the RD and the intersection of the free surface with the extended RD plane[13]. In this work, the deformation modelling considers the 2018 mean displacement velocity prior to the sector collapse. As the tracks provide different viewing geometries (ascending and descending), the method is sensitive to both vertical and horizontal displacements. No further weighting was applied. We implement the RD in a non-linear inversion scheme based on the genetic algorithm (GA)[17], where the objective function (OF) that is minimized is the L2-norm of the model residuals. The solution space chosen was initially wide (dimension was free, plunge 0 degrees, dip from 0 to 90 degrees, strike from 0 to 360 degrees, rake from -100 to -80 degrees). We define a GA population size of 60, a mutation rate of 0.15, and 1000 iterations, with a Poisson ratio of 0.25. **c** Residuals created by calculating the difference of the data and the model. Largest absolute residual values exceed 10 cm in those regions of young material depositions in the southern sector of the island. We examined this feature and conjecture that there some signal may arise from compaction or cooling of young volcanoclastic material. Independent tests have been made where we were masking out the southern sector, but found generally comparable results for the RD source, implying that the new material addition in the southern sector of the island had minor effect only on the inversion.

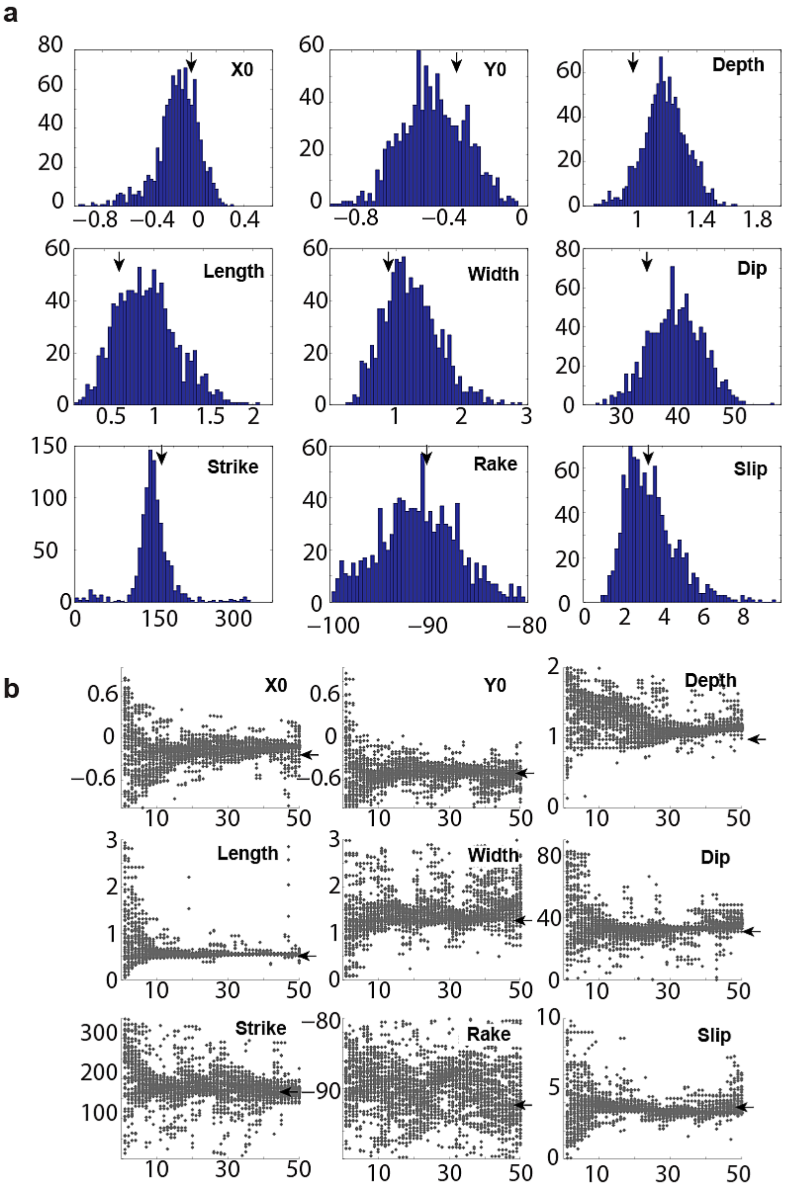

Supplementary Figure 7. Optimization results for rectangular dislocation. a Search parameter variance and **b** parameter conversion during the inversion. The black arrow shows the final solution of the optimization. The EW position is defined relative to the volcano center. The inferred sliding plane has dimensions of 0.48 x 1.24 km^2, located at 0.85 km depth, striking NW-SE at 162 degrees, and dipping to the SW at 34 degrees. Cumulative slip along this fault is 3.3 m for the period Jan 2018-Dec 2018.

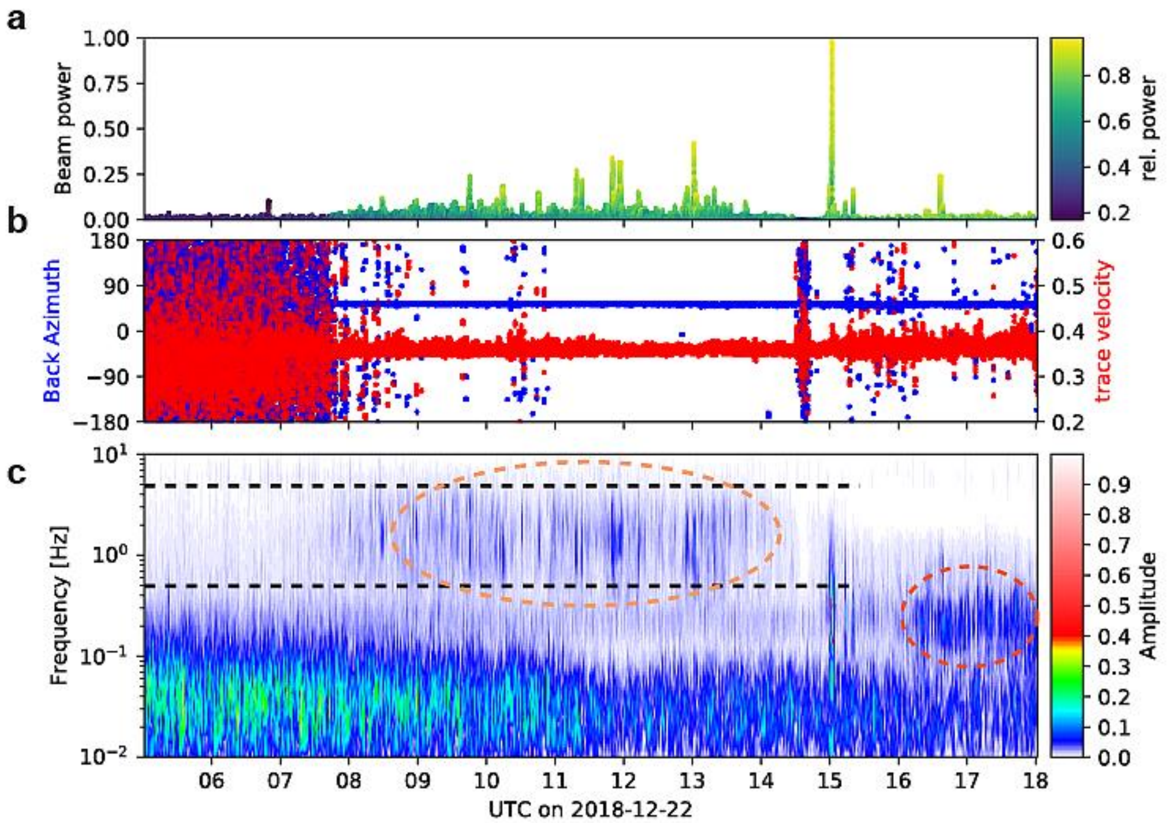

Supplementary Figure 8. Analysis of the infrasound array data recorded at station I06AU of the IMS of the CTBO (12.1491°S, 96.8221°E, distance from Krakatau 1158.8 km) in the hours before the sector collapse reveals that Anak Krakatau was already in an elevated state. **a** Beam power of infrasound station I06AU. **b** Azimuth and trace velocity of the beam. **c** Spectrogram of the beam. Black dashed lines mark the frequency band used for beam-forming. Orange and red dashed lines mark continuous energy release from Krakatau before and after the main event, respectively. We note that approximately one hour before the sector collapse, the activity is markedly reduced, even falling to levels insufficient to form a coherent beam for approximately 15 minutes (from ~14:35 arrival time). Eye-witness accounts support these findings: the activity increased to a subjective peak 2-3 hours before the sector collapse at 11:30 UTC, and when new materials deposited a strong red glow formed, with the eruption turning more ash rich by 12:00 UTC. During the half hour before the sector collapse (13:30-14:00 UTC), the eruption sounds stopped, just before the tsunami was triggered. More detailed visual descriptions can be found on the following website (accessed on 4 March 2019): http://www.oysteinlundandersen.com/krakatau-volcano-witnessing-the-eruption-tsunami-22december2018/

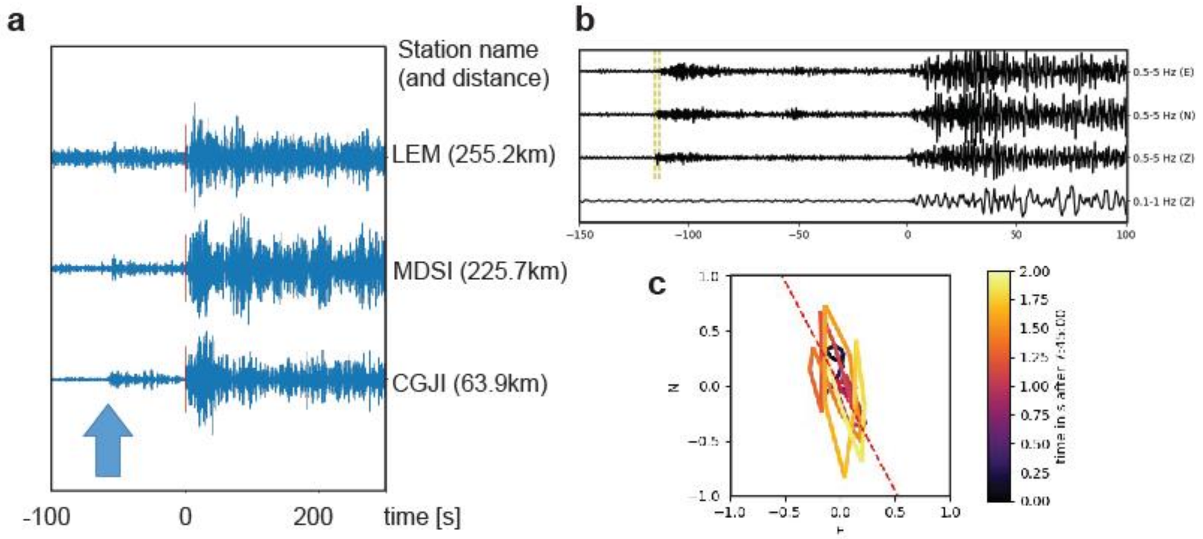

Supplementary Figure 9. High-frequency event. a Prior to the main tremor event, we observed a high-frequency event at Krakatau (marked by the arrow). The timing is 115 s prior to the main event and is observable in the 1-2 Hz frequency band at multiple stations (CGJI, MDSI and LEM shown here), which are situated at three different azimuths (332°, 142° and 288°, respectively). At high frequencies (>1 Hz), the main event (landslide) is clearly visible, here aligned at t=0. Only vertical components are plotted here. Note that the noise level after the precursor stays elevated with respect to the state before the precursor signal, indicating that this is not an ordinary tectonic event. **b** Polarization of the horizontal components of the P-wave. For polarization analysis, data are filtered in the 0.5-5 Hz frequency band and the horizontal components are plotted with respect to each other. The window is chosen around the maximum on the Z-component (yellow dashed lines). **c** The polarization approximately fits the direction towards Krakatau (332°, red dashed line). A comparison of amplitudes of the precursor event and the main event after convolution with a Wood-Anderson instrument response at CGJI yields a difference in M_L of 0.6, implying an estimated M_L of 2.5 for the precursor event. As this estimate is based on a single station, its uncertainty is quite large (±0.5 based on typical scatter of magnitude estimates at this range).

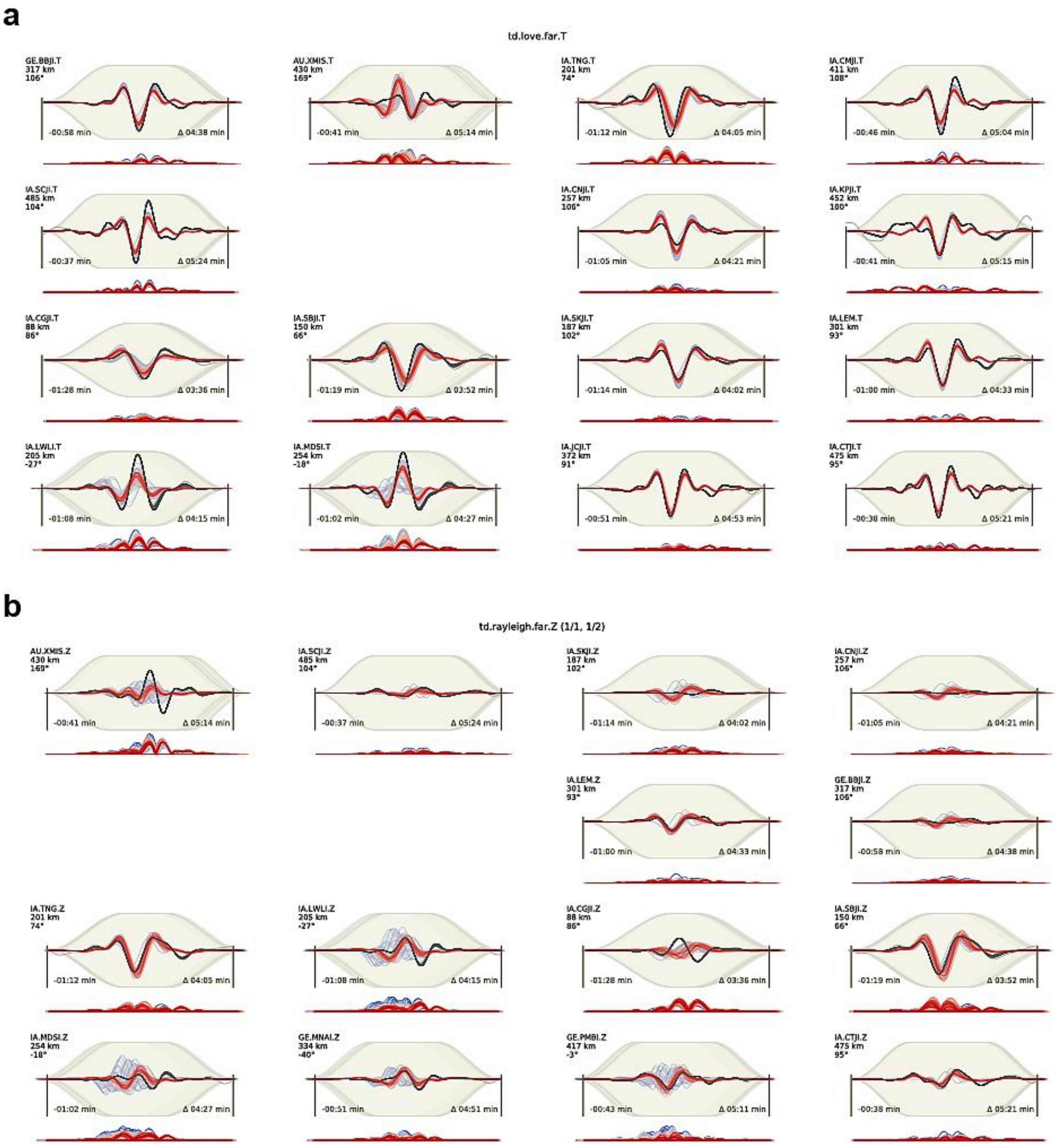

Supplementary Figure 10. Ensemble fit of seismograms. a Ensemble fit of Love wave seismograms (transversal component) filtered between 0.01 and 0.03 Hz. Black lines show observed ground displacement data. Colored traces show simulated waveforms for the ensemble of best centroid moment tensor models, where red and blue indicate smaller and larger misfit solutions, respectively. The lines at the bottom represent the corresponding absolute residuals. Scaling of the seismograms and residuals is performed taking into account station weighting factors to make the visual amplitudes reflect their relative importance in the combined misfit. Station information and window length are indicated by labels. Gray shaded areas represent the taper used to cut out the observed waveforms. **b** Ensemble fit of Rayleigh wave seismograms (vertical component).

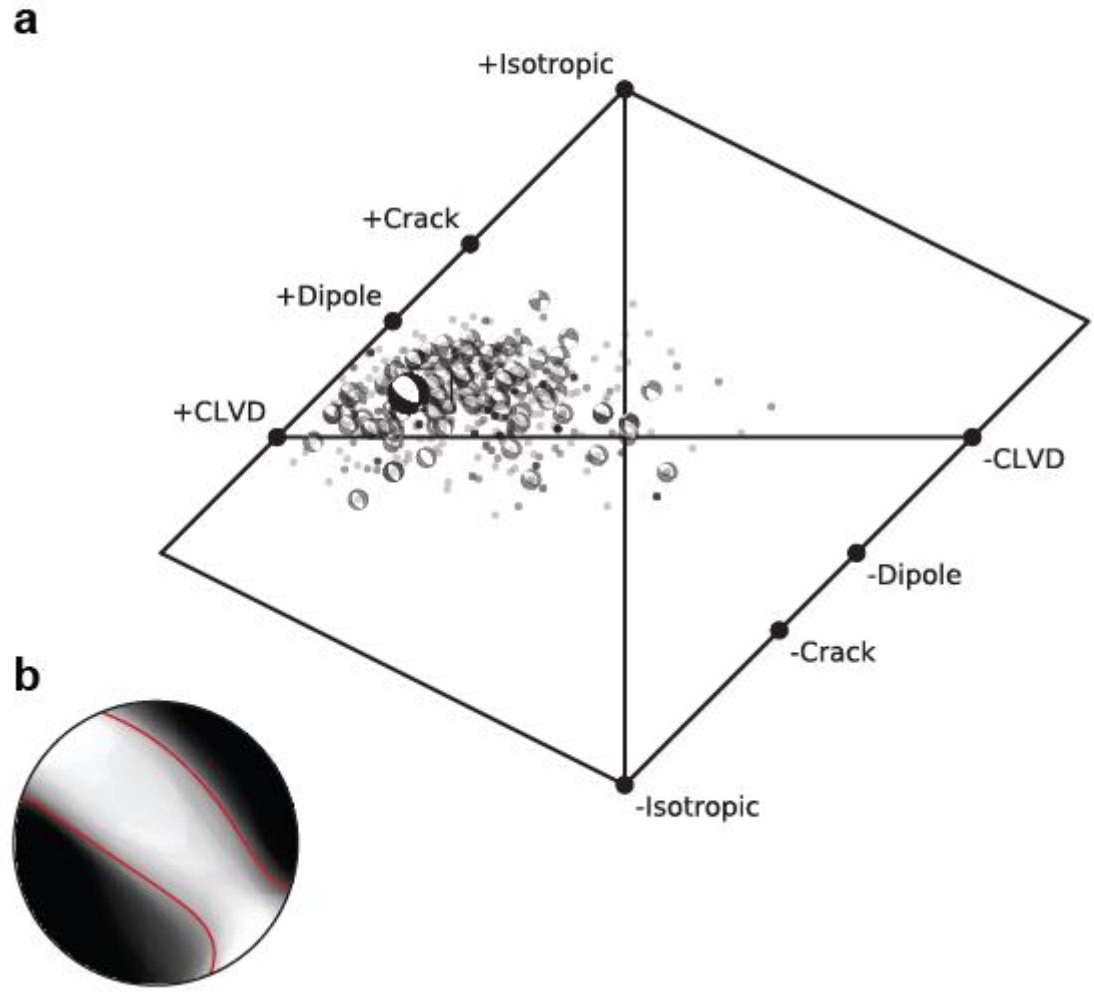

Supplementary Figure 11. Mechanism of faulting/landsliding. a Hudson's source type plot with the ensemble of best fitting moment tensor bootstrap solutions (small symbols) and the model with the lowest misfit (large symbol). The Hudson plot depicts the decomposition of a moment tensor into isotropic, CLVD and DC components under the constraint that the eigenvectors of the CLVD and the DC components are aligned. The inlay focal sphere diagrams depict the DC component of the moment tensors. **b** Fuzzy focal sphere representation of the ensemble of best fitting moment tensor bootstrap solutions. Individual solutions (deviatoric component) are stacked in this representation to visualize the uncertainty of the result. The dominant T-axis of the solution (positive dipole) has an azimuthal direction of 222° and a dip of 12°. Inferred DC fault planes are therefore described by the following parameters: Plane 1: strike1 = 112.991, dip1 = 60.7881 to the SW, rake1 = -117.259; Plane 2: strike2 = 339.544, dip2 = 39.1145 to NE, rake2 = -50.678. The SW dipping plane is in agreement to the plane inferred from InSAR analysis shown in Supplementary Figure 6.

a

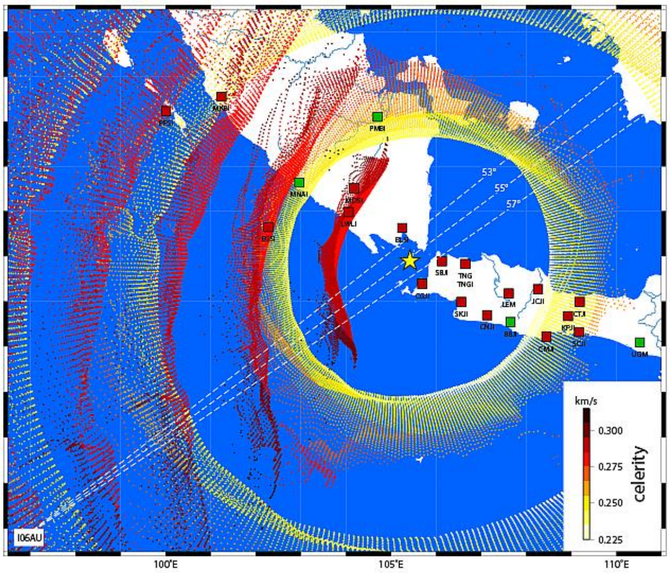

b

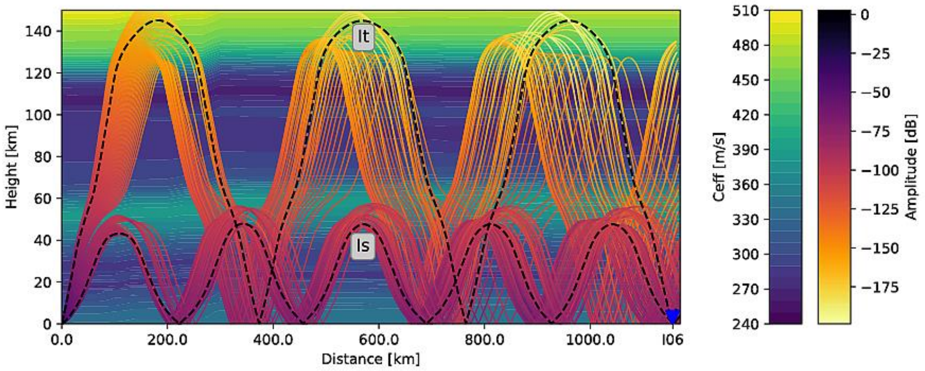

c

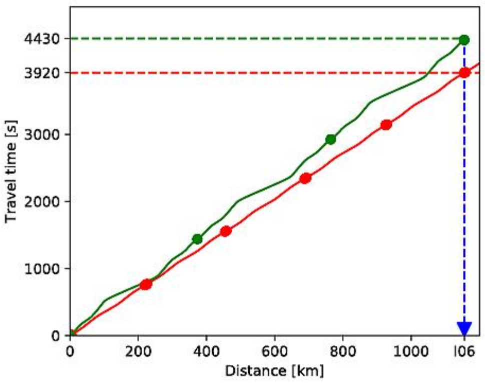

Supplementary Figure 12. Infrasound records. a Bounce-point distribution from rays starting at Krakatau. The infrasound wave was clearly identified at the infrasound array I06AU (southwestern corner of map), but an infrasound phase of the main event was also identified at seismic stations LWLI and MDSI. Networks GE and IA are indicated with red and green symbols, respectively. **b** Infrasound modeling from Krakatau towards infrasound station I06AU (marked with a blue triangle). Eigenrays connecting Krakatau with I06AU are marked with dashed black lines. The effective velocity (speed of sound + wind in the respective direction, as estimated by combining the ECMWF forecast model for altitudes lower than 77 km with HWM14 and NRLMSIS00 for higher altitudes) is derived along the great circle path connecting Krakatau and I06AU and plotted as background colors. **c** Travel times of stratospheric (red) and thermospheric (green) eigenrays (marked as dashed black lines in **b**) as a function of distance. Surface bounces are marked as points. At the location of I06AU at a distance of 1155.7 km, both eigenrays reach the surface after a travel time of 3920 ±4 and 4430 s, respectively. The recordings suggest that only the stratospheric ray path yields sufficient amplitudes to form a coherent beam.

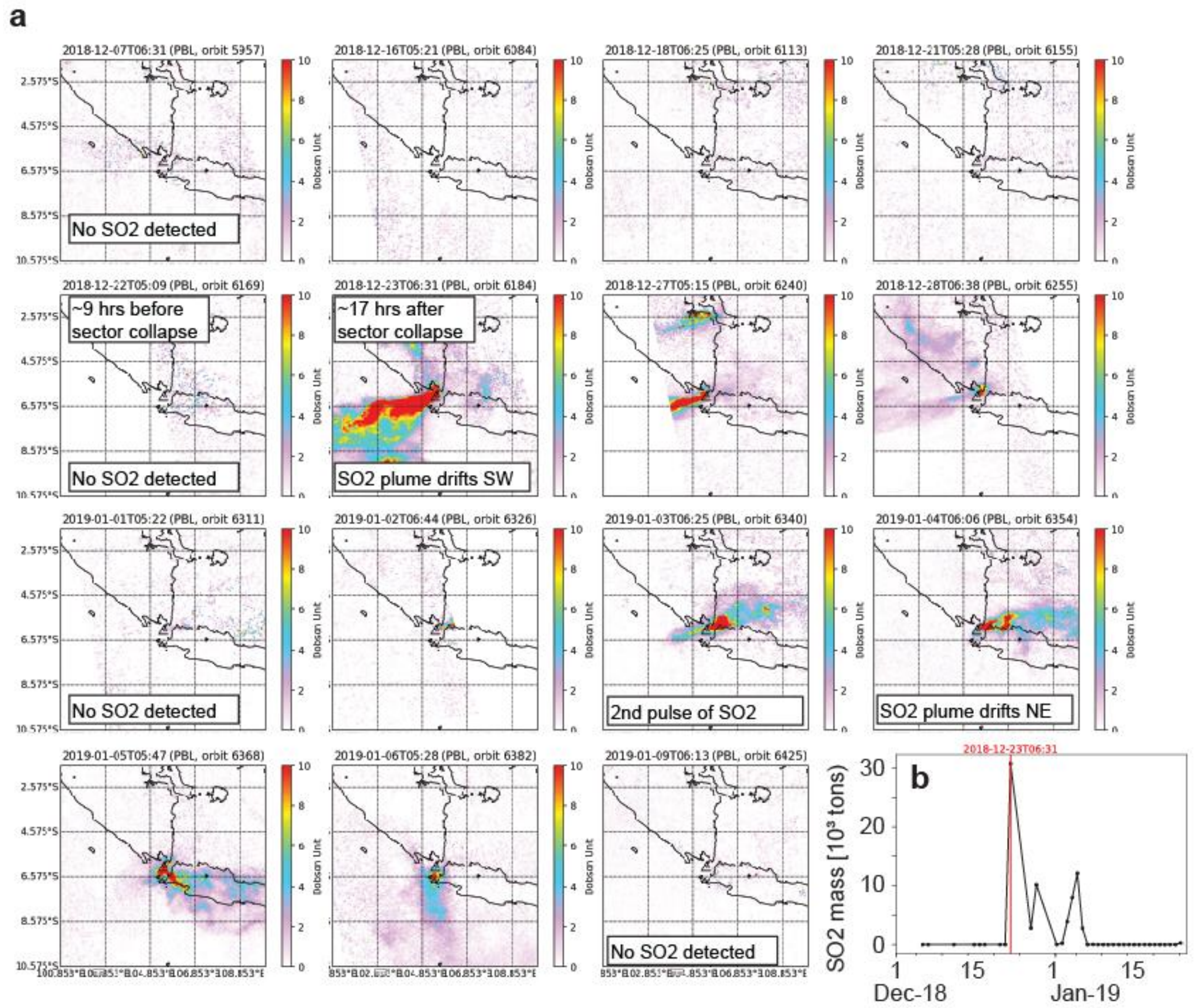

Supplementary Figure 13. SO2 emission. Gas emissions were monitored using remote sensing data acquired by the European Sentinel 5P satellite. **a** The SO_2 emission activity at Anak Krakatau was very low before the collapse, commonly below the detection limit of the sensor, whereas a large anomaly appeared after the collapse. On the day of the flank collapse, poor weather conditions inhibited any view of the volcano and its degassing. The first strong gas plume, drifting to the SW, was identified hours after the collapse when the first clear image was acquired. After a quiescence phase, a second plume, drifting to the NE, was identified in early January 2019. **b** Time series data show that directly after the flank collapse, the SO_2 emissions sharply increased.

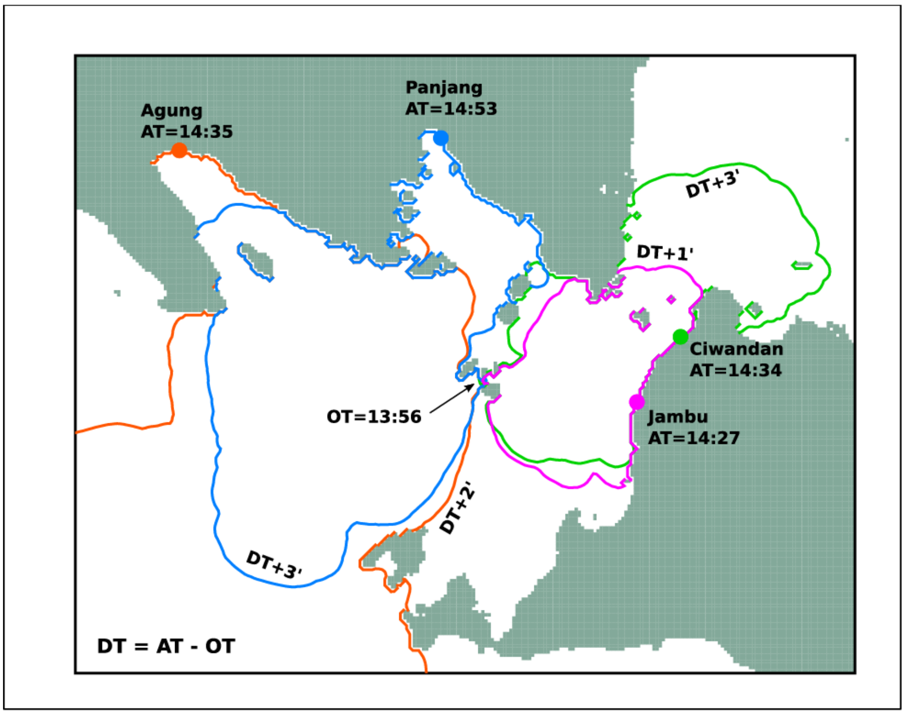

Supplementary Figure 14. Tsunami travel time backtracing. The isochrons show all possible tsunami source points for each station at approximately 13:56 (the seismically determined onset time of the slope instability). The isochrons intersect at Anak Krakatau. It was necessary to shift the picked arrival times by 1-3 minutes to make all four isochrons overlap and agree with the seismic origin time (marked as DT+x in the figure). Given that a coarse global bathymetry was used, this small discrepancy is expected (see Methods for more detail) AT: arrival time, OT: (seismic) origin time.

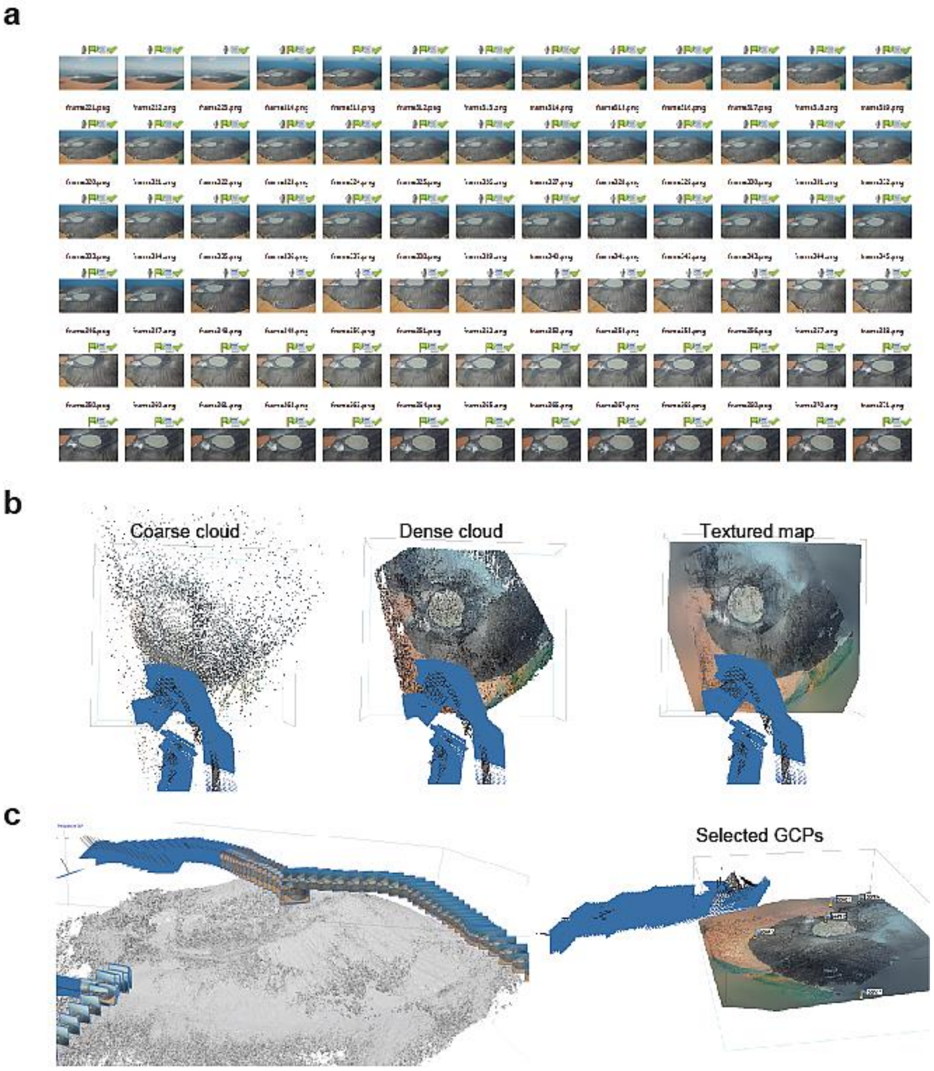

Supplementary Figure 15. Drone video data processing for digital surface model extraction. Drone flights were performed from a fishing vessel outside the 5 km exclusion zone on January 9-10, 2019, i.e., 19 days after the tsunami. To map the surface of Anak Krakatau, we used a camera quadrocopter drone (DJI Mavic Pro), which is GPS controlled and is equipped with a high-quality camera that recorded 4K videos (3840 x 2178) at 30 fps (frames per second), using a cropped 1/2.3-inch CMOS sensor with a 28 mm lens and a field of view of 78.8° at f/2.2, stabilized by a 2-axis gimbal to reduce the vibration artifacts common to quadcopters. **a** Image quality estimate and selection. Due to the large flight distance from the boat positioned to the southeast of Rakata (the greater Krakatau island), the best available video footage was retrieved from the southern side of the Anak Krakatau. These drone flights yielded six 4K videos at high altitudes that we used for photogrammetric processing. From these videos we extracted 12,000 images, and identified the 450 images with the best image clarity, field of view, 90% overlap, 70% sidelap. **b** Algorithm workflow showing the coarse cloud, the dense cloud and the textured model of Anak Krakatau, performed with the Structure from Motion and Multi-View Stereo (SfM-MVS) algorithms included in the Agisoft Metashape Pro software package (version 1.5.0). The images were photogrammetrically aligned so that a dense point cloud composed of ~2,630,000 points could be retrieved by identifying 16,000 tie points. Direct Ground Control Points (GCPs) could not be acquired due to the exclusion zone and hazardous access to the volcano. Therefore we follow the strategy[18] by using the internal GPS of the drone for coarse location, then co-align the three-dimensional point cloud to an independent dataset of a known quality. A cloud comparison to TanDEM-X topographic data was realized using the point pair-picking registration in the CloudCompare software (version 2.9.1) [GPL software]. This yielded a reprojection error of 8.9 pixels. The vertical mean error of TanDEM-X data is smaller than 0.2 m, and has an Root Mean Square Error (RMSE) smaller 1.4 m [19]. The resulting DEM has a resolution of 0.4 to 1.3 m, which we resampled to 1 m. Further data handling was performed using GISanalysis (ArcMap 10.2.1). **c** The flight path was from the southeast, and the images are shown by blue quadrangles; the reconstructed point cloud quality is highest in the southeastern sector of Anak Krakatau.

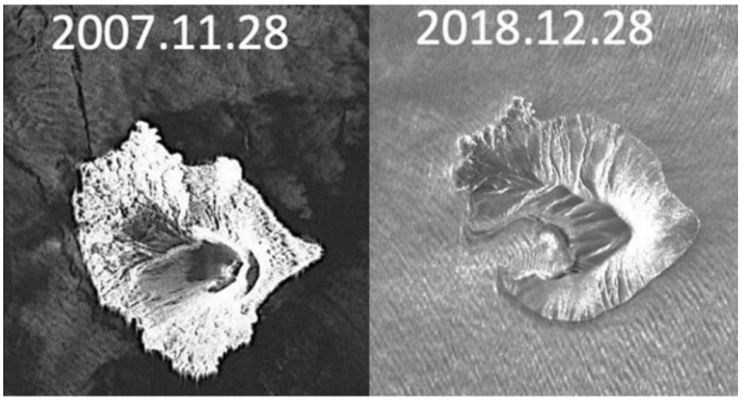

Supplementary Figure 15. Material loss and gain. We compare the topography derived from the drone images to the DEM from the TanDEM-X satellite mission. The difference between the DEMs yields a volume change of ~0.1 km^3. However, we believe that this number significantly underestimates the true volume for a number of reasons, mainly due to (i) the timing of the pre-eruption DEM, (ii) the timing of the post-eruption DEM, and (iii) the lack of submarine information. Quantification of the collapsed volume from our dataset is therefore difficult. The topographic dataset available for the precollapse episode is the global TanDEM-X DEM. While this DEM is of a very high quality and unprecedented in precision, it was released in 2016 [19] and therefore does not consider recent activity and lacks the volume added by lava flow emissions (such as in the period July-December 2018). Therefore, this DEM underestimates the real dimensions of Anak Krakatau. The postcollapse DEM is constructed from our own drone overflight data acquired on 9-10 January 2019, i.e., 18-19 days after the sector collapse. Consequently, this material does consider the volume loss resulting from the collapse but does not consider the deposition of new tephra associated with the eruptions during the period 22 Dec 2018 – 9 Jan 2019. As a result, this DEM might underestimate the volume loss and/or overestimate the dimensions of Anak Krakatau immediately after the collapse. The coeruptive tephra deposition is best seen on the northern and eastern slopes of the edifice, where the coastline shifted by 120 to 260 m northeastwards, which may represent a volume gain in the northeastern sector of the island of 3.17x10^7 m^3 (assuming one-fourth of a frustum of a cone in the northeast, a height of 140 m and radius change from 620 to 820 m). Further details for the northernmost, northwestern and western parts of the island cannot be provided because the drones did not fly into these areas, but it can by hypothesized that in the other sectors accumulated a similar amount of tephra (which would represent a volume change on 0.13 km^3). The submarine volume of the landslide cannot be assessed and was beyond the scope of this work. From our investigation of the satellite imagery, we assume that a rather large submarine portion may have been involved in the landslide, similar to that previously proposed by [20]. Shortly after the collapse, the morphology and bathymetry profoundly changed, as the eruptions immediately occurring after the 22 Dec 2018 collapse resulted in substantial rebuilding of the tuff cone and crater lake, and the flanks of Anak Krakatau expanded. This submarine change can be inferred from the sequence of radar imagery in the first two weeks after the collapse and requires consideration in forthcoming studies. Therefore, we conclude that appropriate measures of the volume of the collapse cannot be made using the dataset presented. The 0.1 km^3 material loss estimated from simple DEM subtraction is very conservative and could increase to 0.23 km^3 if the tephra deposition is considered. This number is still conservative and may have been accompanied by a submarine loss of the same order of magnitude.

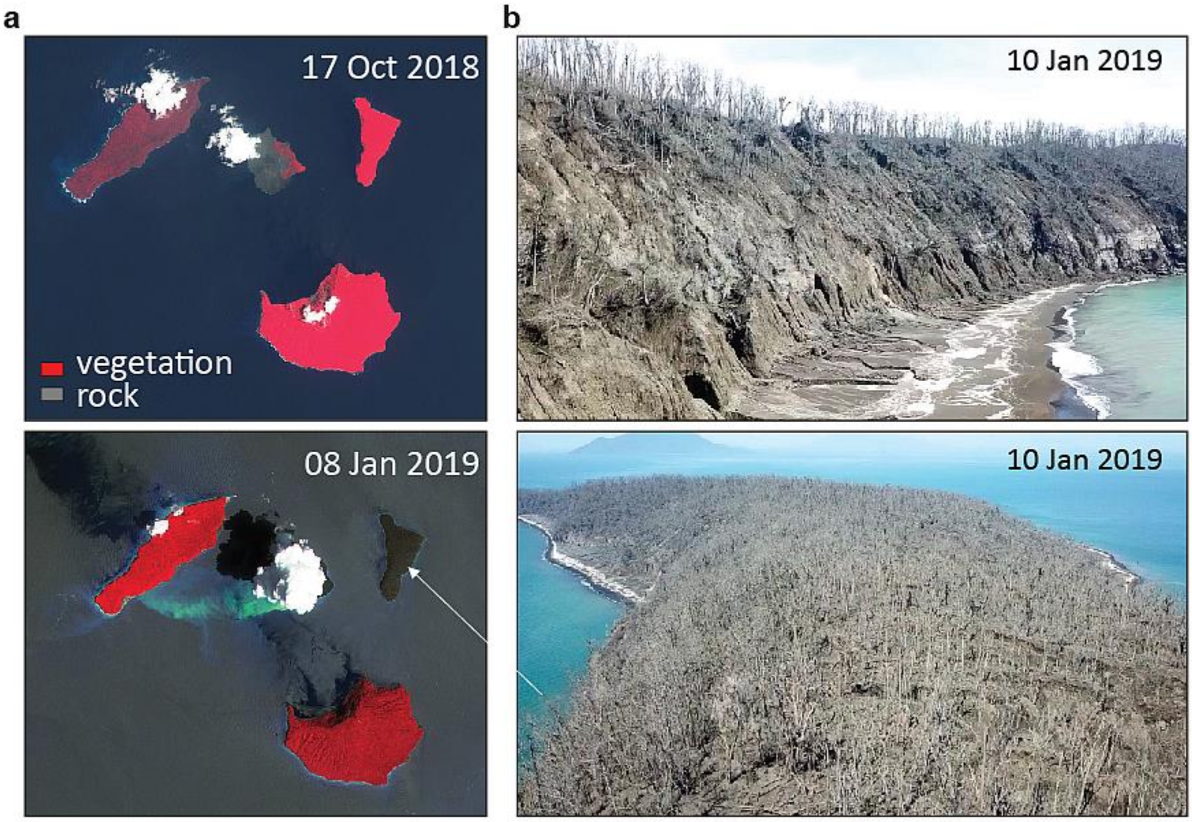

Supplementary Figure 16. Destruction over the island as identified in Sentinel 2 data and as seen from drone photos. **a** Sentinel-2 bands 8, 4, and 3 in combination with the RGB channels show vegetation as red and loss of vegetation after eruption in dark gray. **b** The drone images reveal the profound destruction on the adjacent island of Kecil, most likely associated with devastation due to surge and gas plumes. The upper image shows coastline erosion on western Kecil. Lower image shows view over Kecil, looking to the north.

Table. XRF data for recent Anak Krakatau eruptives

Sample	KR-1	KR-3	KR-5	KR-6	KR-7	KR-9	KR-10	KR-11	KR-12	KR-13
	Ash July 2018	Lava	Lava	Lava	Lava	Lava	Lava collected Dec 2010	Rec. Lava	Rec. Lava	Rec. Lava
SiO2(%)	53.42	54.18	54.95	53.63	54.03	54.64	54.61	54.48	54.97	54.18
TiO2(%)	1.12	1.11	1.09	1.13	1.11	1.10	1.10	1.12	1.11	1.12
Al2O3(%)	16.95	17.43	17.63	16.92	17.33	17.33	17.25	17.45	17.64	17.18
MnO(%)	0.166	0.164	0.162	0.165	0.166	0.162	0.163	0.163	0.165	0.165
MgO(%)	4.17	4.09	3.63	4.13	3.90	3.54	3.56	3.53	3.58	3.63
CaO(%)	8.09	8.29	7.95	8.17	8.15	7.92	7.91	7.94	7.96	7.94
Na2O(%)	3.58	3.79	4.07	3.64	3.77	3.87	3.85	3.88	4.03	3.84
K2O(%)	0.88	0.88	0.95	0.90	0.90	0.97	0.97	0.94	0.93	0.93
P2O5(%)	0.238	0.238	0.256	0.239	0.244	0.250	0.249	0.250	0.258	0.251
T-Fe2O3(%)	9.39	9.39	9.02	9.17	9.21	8.89	8.94	9.08	9.11	9.18
H2O(%)	0.06	0.06	0.09	0.07	0.03	0.07	0.00	0.11	0.04	0.07
Total(%)	98.06	99.50	99.80	98.26	98.84	98.74	98.6	98.94	99.79	98.49
Ba(ppm)	137.20	124.00	137.10	134.00	126.6	142.80	159.90	140.20	138.2	132.30
Ce(ppm)	34.10	29.50	32.00	31.90	31.10	33.60	38.40	32.00	32.6	30.50
Co(ppm)	42.26	52.54	52.54	55.25	57.86	52.06	52.70	52.54	50.87	50.02
Cr(ppm)	26.94	22.33	13.71	18.49	21.43	14.16	19.31	16.17	22.44	24.66
Cu(ppm)	20.10	19.30	13.00	21.30	19.70	18.80	20.80	18.5	14.30	19.50
Dy(ppm)	4.32	4.25	4.19	4.27	4.25	4.17	4.19	4.24	4.23	4.26
Ga(ppm)	18.00	18.00	18.1	18.10	18.00	18.10	18.20	18.10	18.10	18.00
Hf(ppm)	1.96	1.84	2.29	2.46	2.28	2.78	3.05	3.05	2.50	2.05
Nb(ppm)	4.30	4.30	4.33	4.33	4.49	4.61	4.29	4.51	4.46	4.50
Nd(ppm)	16.00	15.65	15.83	15.76	15.75	15.98	16.42	15.81	15.85	15.63
Ni(ppm)	4.16	3.28	2.13	4.94	3.42	1.55	3.25	1.98	2.49	0.72
Pb(ppm)	9.93	9.85	9.77	9.83	9.70	9.99	10.32	9.96	9.97	9.93
Pr(ppm)	6.17	5.95	6.03	6.03	6.02	6.14	6.30	6.06	6.09	6.00
Rb(ppm)	22.58	22.03	23.40	22.59	22.33	24.34	25.26	23.93	23.75	23.27
Sc(ppm)	32.21	32.04	30.31	32.32	31.49	31.38	30.12	30.67	30.25	31.61
Sr(ppm)	332.90	336.20	338.40	340.20	333.70	335.10	337.40	339.80	341.30	336.8
Th(ppm)	<LLD	<LDD	<LDD	<LDD	<LDD	<LDD	<LDD	<LDD	<LDD	<LDD
U(ppm)	0.94	0.76	0.86	0.83	0.76	0.98	1.21	0.96	0.99	0.89
V(ppm)	208.30	207.30	203.30	208.30	207.10	202.20	203.40	204.90	206.30	209.60
Y(ppm)	19.70	19.60	20.30	20.10	19.90	21.00	21.70	20.60	20.50	20.30
Yb(ppm)	3.14	4.12	4.77	4.90	5.03	4.94	5.22	4.47	4.24	4.12
Zn(ppm)	80.80	80.00	79.60	81.10	80.50	80.40	81.40	81.20	80.50	80.80
Zr(ppm)	104.90	104.70	109.90	106.80	106.10	113.10	114.70	110.90	110.70	109.50

Samples were analysed by XRF at BPPTKG, Yogyakarta, Indonesia (report number 02/LHU-XRF/2019).

Supplementary Table 1. Whole rock geochemical analysis of 2018 eruptive products. See also Supplementary Figure 3.

Month	Area (km^2)
January	2.93
February	2.94
March	2.95
April	2.94
May	2.94
June	2.96
July	2.96
August	3.00
September	3.01
October	3.05
November	3.02
December	3.03
January	3.39

Supplementary Table 2. Island area increase derived from SAR amplitude images and edge detection (see Supplementary Figure 3). Monthly island area in 2018 and January 2019 was calculated from the coastline shapefiles.

Dataset	Path number	Number of images	Time span	Heading angle (°)	Incidence angle (°)
S-1 Ascending	171	30	5 Jan 2018-<br>19 Dec 2018	-12	43.1
S-1 Descending	47	29	20 Jan 2018-<br>08 Dec 2018	192	44.9
S-1 Descending	120	29	1 Jan 2018-<br>15 Dec 2018	192	31.4

Supplementary Table 3. Details of Sentinel-1 SAR data used in InSAR time series analysis

Starting condition	Inversion:
X position [km]: -1 to 1	-0.111
Y position [km]: -1 to 1	-0.373
Depth [km]: 0 to 2	0.855
Length [km]: 0.01 to 3	0.479 (horizontal RD edge)
Width [km]: 0.01 to 3	1.239 (down-dip RD edge)
Plunge [deg]: 0 to 0	0
Dip [deg]: 0 to 90	34.851
Strike [deg]: 0 to 360	162.822
Rake [deg]: -100 to -80	-90.509
Slip [deg]: 0 to 10	3.3578

Supplementary Table 4. Source parameters derived from inversion of InSAR deformation data

Supplementary References

1. Bredemeyer, S., Ulmer, F. G., Hansteen, T. H. & Walter, T. R. Radar Path Delay Effects in Volcanic Gas Plumes: The Case of Lascar Volcano, Northern Chile. *Remote Sens-Basel* **10**, doi:10.3390/Rs10101514 (2018).
2. Meyer, F. J. *et al.* Integrating SAR and derived products into operational volcano monitoring and decision support systems. *Isprs Journal of Photogrammetry and Remote Sensing* **100**, 106-117, doi:10.1016/j.isprsjprs.2014.05.009 (2015).
3. Gorelick, N. *et al.* Google Earth Engine: Planetary-scale geospatial analysis for everyone. *Remote Sens Environ* **202**, 18-27, doi:10.1016/j.rse.2017.06.031 (2017).
4. Dahren, B. *et al.* Magma plumbing beneath Anak Krakatau volcano, Indonesia: evidence for multiple magma storage regions. *Contrib Mineral Petr* **163**, 631-651, doi:10.1007/s00410-011-0690-8 (2012).
5. Gardner, M. F. *et al.* Crustal Differentiation Processes at Krakatau Volcano, Indonesia. *J Petrol* **54**, 149-182, doi:10.1093/petrology/egs066 (2013).
6. Hooper, A. A multi-temporal InSAR method incorporating both persistent scatterer and small baseline approaches. *Geophys Res Lett* **35**, doi:10.1029/2008gl034654 (2008).
7. Motagh, M. *et al.* Quantifying groundwater exploitation induced subsidence in the Rafsanjan plain, southeastern Iran, using InSAR time-series and in situ measurements. *Eng Geol* **218**, 134-151, doi:10.1016/j.enggeo.2017.01.011 (2017).
8. Aryal, A., Brooks, B. A. & Reid, M. E. Landslide subsurface slip geometry inferred from 3-D surface displacement fields. *Geophys Res Lett* **42**, 1411-1417, doi:10.1002/2014GL062688 (2015).
9. Savage, W. Z. & Chleborad, A. F. A model for creeping flow in landslides. *Bull. Assoc. Eng. Geol.* **19**, 333-338 (1982).
10. Muller, J. R. & Martel, S. J. Numerical models of translational landslide rupture surface growth. *Pure Appl Geophys* **157**, 1009-1038, doi:DOI 10.1007/s000240050015 (2000).
11. Booth, A. M., Lamb, M. P., Avouac, J. P. & Delacourt, C. Landslide velocity, thickness, and rheology from remote sensing: La Clapiere landslide, France. *Geophys Res Lett* **40**, 4299-4304, doi:10.1002/grl.50828 (2013).
12. Nikolaeva, E., Walter, T. R., Shirzaei, M. & Zschau, J. Landslide observation and volume estimation in central Georgia based on L-band InSAR. *Nat Hazard Earth Sys* **14**, 675-688, doi:10.5194/nhess-14-675-2014 (2014).
13. Nikkhoo, M., Walter, T. R., Lundgren, P. R. & Prats-Iraola, P. Compound dislocation models (CDMs) for volcano deformation analyses. *Geophys J Int* **208**, 877-894, doi:10.1093/gji/ggw427 (2017).
14. Comninou, M. & Dundurs, J. The angular dislocation in a half space. *J. Elast.* **5**, 203-216 (1975).
15. Okada, Y. Surface deformation due to shear and tensile faults in a half-space. *B Seismol Soc Am* **75**, 1135-1154 (1985).
16. Okada, Y. Internal deformation due to shear and tensile faults in a half-space. *Bull. Seism. Soc. Am.* **82**, 1018-1040 (1992).
17. Haupt, R. L. & Haupt, S. E. *Practical Genetic Algorithms*. Vol. 2 (Wiley-Interscience, 2004).
18. Muller, D. *et al.* High-Resolution Digital Elevation Modeling from TLS and UAV Campaign Reveals Structural Complexity at the 2014/2015 Holuhraun Eruption Site, Iceland. *Front Earth Sci* **5**, doi:10.3389/Feart.2017.00059 (2017).
19. Wessel, B. *et al.* Accuracy assessment of the global TanDEM-X Digital Elevation Model with GPS data. *Isprs J Photogramm* **139**, 171-182, doi:10.1016/j.isprsjprs.2018.02.017 (2018).
20. Giachetti, T., Paris, R., Kelfoun, K. & Onotowirjo, B. Tsunami hazard related to a flank collapse of Anak Krakatau. *Geological Society, London, Special Publications* **361**, 79-90, doi:10.1144/SP361.7 (2012).
